# Acute Pain Transfusion Reaction: A Case Report

**DOI:** 10.7759/cureus.64206

**Published:** 2024-07-10

**Authors:** Purshotam Paudel, Pammy Sinha

**Affiliations:** 1 Transfusion Medicine, Sri Lakshmi Narayana Institute of Medical Sciences, Pondicherry, IND; 2 Pathology, Sri Lakshmi Narayana Institute of Medical Sciences, Pondicherry, IND

**Keywords:** packed red blood cells ( prbc), transfusion reaction, pain, cytokines, acute pain transfusion reaction

## Abstract

Transfusion-related adverse events involving packed red blood cells (PRBCs) and fresh frozen plasma (FFP) are not unusual. Reactions can happen at any time during the transfusion, as well as hours or days later. An acute pain transfusion reaction (APTR) is defined as sudden, intense joint pain, usually in the back and trunk, that appears right after transfusion after all other potential causes of transfusion reactions have been eliminated. The present article discusses two similar cases. A 38-year-old female presented with complaints of right-sided headache and photophobia for four days, associated with nausea, vomiting, and vertigo. She was evaluated for a migraine headache. Due to anemia, a one-unit PRBC was requested. After pre-transfusion testing, a one-unit non-leuko-reduced, coombs cross-match compatible B-positive packed red blood cell (PRBC) was issued and transfused. During the transfusion, the patient complained of chest pain. The transfusion was stopped. Her vitals did not vary much from the baseline. No other symptoms were present at that time.

A 69-year-old female presented with complaints of vomiting, abdominal pain, and black tarry stool for a one-month duration. On evaluation, she was diagnosed with adenocarcinoma of the stomach. Given the increased prothrombin time/international normalized ratio (PT/INR) of 1.8, four-units of fresh frozen plasma (FFP) was requested, which was issued after performing minor cross-match compatibility. After five minutes of transfusion, she complained of severe pain at the transfusion site with chills and rigors. The transfusion was stopped. There was no change in the vitals of the patient from baseline. A complete workup was done to rule out other transfusion reactions in both cases. Thus, these patients experienced what is known as an acute pain transfusion reaction. APTR is typically self-limited and requires treatment of symptoms with pain control, supplemental oxygen, and emotional support. In both cases, supportive treatments were enough to control the pain symptoms of the patients.

## Introduction

Transfusion reactions are defined as adverse events associated with the transfusion of whole blood or one of its components. These may range in severity from minor to life-threatening. These can be immunological or non-immunological. Reactions can happen during transfusion (acute reaction) or days to weeks later (delayed transfusion reactions), up to 28 days. A reaction might be difficult to detect since it can cause nonspecific, frequently overlapping symptoms [1]. The most common signs and symptoms are fever, chills, urticaria (hives), and itching. Some symptoms improve with minimal or no treatment. However, respiratory discomfort, high temperature, hypotension, and red urine (hemoglobinuria) may signal a more serious reaction.

Transfusion-related adverse events involving packed red blood cells (PRBCs) and fresh frozen plasma (FFPs) are not unusual. An acute pain transfusion reaction (APTR) is defined as sudden, intense joint pain, usually in the back and trunk, that appears right after transfusion after all other potential causes of transfusion reactions have been excluded. Additional clinical characteristics that some individuals may have include dyspnea, tachycardia, hypertension, and pain that is limited to the transfusion-related limb [2]. Here we present two similar kinds of cases that we encountered in our center.

## Case presentation

Case 1

A 38-year-old female presented with complaints of right-sided headache and photophobia for four days, associated with nausea, vomiting, and vertigo. She was evaluated in the line of migraine headache. Her initial investigations revealed a hemoglobin level of 5.4 g/dl, a white blood cell (WBC) count of 14100/uL, and a platelet count of 3.1 lakh. Due to anemia, a one-unit PRBC was requested. After pre-transfusion testing, a one-unit non-leuko-reduced, coombs cross-match compatible saline adenine glucose mannitol (SAGM) PRBC B-positive was issued at 3:00 p.m. The transfusion started at 3:45 p.m. During the transfusion at 7:10 p.m., the patient complained of chest pain. The transfusion was stopped. Her vitals did not vary much from the baseline. There was no fever, chills, dyspnea, shortness of breath, discoloration of urine, or loin pain. Her vitals were as follows: blood pressure (BP): 120/80 mm Hg; pulse: 104 bpm; temperature: 97 °F; and respiratory rate (RR): 20/min. She was kept under close monitoring. After 30 minutes, her condition improved, and the remaining PRBC was transfused as per the clinician’s order. She was not given any medications during the episode of reaction. Following the blood bank policy, a post-transfusion work-up was initiated, including bedside confirmation of the proper product and patient identification. Clerical errors were ruled out. There was no hemolysis in the returned PRBC unit as well as in the post-transfusion sample. The post-reaction patient group matched their pre-transfusion sample with a negative direct antiglobulin test. The repeat grouping of the PRBC unit matched the label. The cross-matching of the patient's pre- and post-reaction samples showed compatibility with the unit. A peripheral blood smear revealed no schistocytes, and serum indirect bilirubin was within the normal range. Therefore, a hemolytic transfusion reaction was ruled out. Other causes of chest pain were ruled out with an electrocardiogram and other investigations.

Case 2

A 69-year-old female presented with complaints of vomiting and abdominal pain for a one-month duration. It was associated with black tarry stool and weight loss. On evaluation, she was diagnosed with adenocarcinoma of the stomach. Blood investigations revealed a hemoglobin of 8.3 g/dl, a WBC of 9800/uL, and a platelet count of 3.2 lakh. Given the increased prothrombin time/international normalized ratio (PT/INR) of 1.8, a four-unit FFP was requested, which was minor cross-match compatible and issued at 2.45 p.m. The transfusion was started at 3 p.m. After five minutes of starting the transfusion, the patient complained of severe pain at the transfusion site with chills and rigors. The transfusion was stopped, and the remaining and other FFP were sent back to the blood bank. Injections of acetaminophen 1 ampoule and diphenhydramine 25 mg were given intravenously. There was no change in the vitals of the patient from baseline. Her vitals were as follows: blood pressure of 130/90 mm Hg; pulse of 90/min; respiratory rate (RR) of 12/min; and temperature of 98.2 F. She was kept under close monitoring. After 30 minutes, her condition improved. Following blood bank policy, a post-transfusion response work-up was initiated, including bedside confirmation of the proper product and patient identification. Clerical errors were ruled out. The post-reaction patient group matched their pre-transfusion sample with a negative direct antiglobulin test. Post-transfusion patients' blood samples revealed total and direct bilirubin of 1 mg/dl and 0.6 mg/dl, and urea and creatinine of 23 mg/dl and 0.7 mg/dl, respectively. The urine microscopic examination was normal, and there was no RBC or cast. There were no other alternative explanations for her pain during the transfusion.

## Discussion

An acute pain transfusion reaction (APTR) is a relatively uncommon and poorly understood transfusion reaction that might develop either during or following a transfusion of blood products. The mechanisms of this rare presentation are poorly understood. It is usually registered in the hemovigilance records as an unclassified reaction when other causes of adverse reactions have been ruled out. Cytokines and leukoreduction filters have been linked to some APTR case reports. However, no underlying causes or mechanisms have been addressed in the limited literature. Patients receiving blood products with a variety of underlying diseases, such as hematologic malignancy, solid tumors, and liver disease, among others, as well as those recovering from surgery, seem to experience this reaction [2]. Our patient's signs and symptoms are similar to those described in the few published case studies on APTR [2-4]. Once all alternative causes of transfusion reactions have been ruled out and negative results from immune hematological and other laboratory work-ups have been made, the diagnosis of APTR should be made. A study conducted by Orton et al. discovered that APTR was contributing to 8% (12 reports) of transfusion reactions in a multi-center retrospective examination of over 29,000 medical records of patients receiving blood transfusions. These twelve cases included the following clinical manifestations: tachypnoea and/or dyspnea, hypertension, tachycardia, and acute pain in the chest, back, or proximal extremities [4].

In both cases, all the immune hematological workup and lab reports were negative to fit into any other transfusion reaction (Table 1). Similarly, there were no other non-transfusion-related causes of chest pain, including cardiac, pulmonary, musculoskeletal, or gastrointestinal causes.

**Table 1:** Post-reaction investigations BG: blood group; DCT: direct coombs test; ICT: indirect coombs test; FFP: fresh frozen plasma; PRBC: packed red blood cells; RBC: red blood cells

Parameters	Case 1	Case 2	Normal Range
Pre-transfusion BG	B Positive	AB Positive	
Post-transfusion BG	B Positive	AB Positive	
Product type	PRBC	FFP	
Blood group of issued products	B Positive	AB Positive	
Post DCT	Negative	Negative	
Post ICT	Negative	Negative	
Cross-match with pre-sample	Compatible	Compatible	
Cross-match with post-sample	Compatible	Compatible	
Total bilirubin	1.2 mg/dl	1 mg/dl	0.2-1.2mg/dl
Direct bilirubin	0.4 mg/dl	0.6mg/dl	0.2-0.4 mg/dl
Peripheral smear	No schistocytes	No schistocytes	
Urine R/E	No RBC cast	No RBC cast	No RBC/cast
Urea	30 mg/dl	23 mg/dl	10-40 mg/dl
Creatinine	0.9 mg/dl	0.7 mg/dl	0.6- 1.4mg/dl

Works of literature have shown that APTR has no propensities for underlying diagnosis. However, it is noted to occur with pre-storage leuko-reduced blood products [2,4,5]. In our first case, it was non-leuko-reduced PRBC; in another case, it was FFP. Few reports have mentioned APTR with PRBC and platelets. However, with FFP, there has not been a report of such a reaction in the literature. In our case, the pain experienced by the patient could be attributed to APTR.

The bulk of transfusion reactions are known to be mediated by cytokines, primarily interleukin (IL)‑1β, IL‑6, IL‑8, and tumor necrosis factor (TNF)‑α, which are regulated by T lymphocytes and released from residual WBC in the blood components. It is unknown how cytokines function in APTR. Remarkably, the majority of APTR case reports that have been reported have happened following the transfusion of leuko-reduced blood products. This suggests that variables other than cytokines could be responsible for the etiology of APTR. In their investigation, Weisbach et al. showed that while IL-1 β and TNF-α levels were considerably decreased by leukoreduction up until the end of the RBC storage period, IL-8 levels did not decrease [6].

In rat models, Cunha et al.’s work showed that IL-8 is a mediator of sympathetic pain [7]. There has been evidence linking human pathological and inflammatory pain to TNFα, IL‑6, and IL‑1 β [8]. In this instance, the fact that the PRBC unit was older than two weeks helps to explain the buildup of cytokines in the unit that resulted in the reaction. However, in the second case, there is no possible explanation for the pain. And our institution does not have the facility to check for interleukin levels. Therefore, more research is needed to determine the causes of this unusual transfusion-associated adverse event as well as its pathophysiology at the cellular and molecular levels. This will help in improving patient care by understanding prevention and treatment options.

## Conclusions

APTR is an infrequently reported event because many times clinicians are unaware of it. This is more true, especially when pain symptoms appear after the completion of a blood transfusion. Therefore, increased awareness is needed among clinicians and transfusion medicine practitioners to recognize and report this rare event. Since these kinds of reactions are usually self-limited and no definitive treatments are available, supportive treatments, including pain control, supplemental oxygen, and emotional support, are needed during such episodes.
